# MiR-214-3p promotes proliferation and inhibits estradiol synthesis in porcine granulosa cells

**DOI:** 10.1186/s40104-020-00500-y

**Published:** 2020-09-14

**Authors:** Shengjie Shi, Xiaoge Zhou, Jingjing Li, Lutong Zhang, Yamei Hu, Yankun Li, Gongshe Yang, Guiyan Chu

**Affiliations:** 1 Key Laboratory of Animal Genetics, Breeding and Reproduction of Shaanxi Province, Yangling, 712100 China; 2 Laboratory of Animal Fat Deposition & Muscle Development, College of Animal Science and Technology, Northwest A&F University, Yangling, 712100 China

**Keywords:** Estradiol synthesis, Granulosa cells, MiR-214-3p, Proliferation

## Abstract

**Background:**

Granulosa cells (GCs) proliferation and estradiol synthesis significantly affect follicular development. The miR-214-3p expression in the ovarian tissues of high-yielding sows is higher than that in low-yielding sows, indicating that miR-214-3p may be involved in sow fertility. However, the functions and mechanisms of miR-214-3p on GCs are unclear. This study focuses on miR-214-3p in terms of the effects on GCs proliferation and estradiol synthesis.

**Results:**

Our findings revealed that miR-214-3p promotes proliferation and inhibits estradiol synthesis in porcine GCs. MiR-214-3p can increase the percentage of S-phase cells, the number of EdU labeled positive cells, and cell viability. However, E_2_ concentration was reduced after miR-214-3p agomir treatment. We also found that miR-214-3p up-regulates the expression of cell cycle genes including cell cycle protein B (*Cyclin B*), cell cycle protein D (*Cyclin D*), cell cycle protein E (*Cyclin E*), and cyclin-dependent kinase 4 (*CDK4*) at the transcription and translation levels, but down-regulates the mRNA and protein levels of cytochrome P450 family 11 subfamily A member 1 (*CYP11A1*), cytochrome P450 family 19 subfamily A member 1 (*CYP19A1*), and steroidogenic acute regulatory protein (*StAR*) (i.e., the key enzymes in estradiol synthesis). On-line prediction, bioinformatics analysis, a luciferase reporter assay, RT-qPCR, and Western blot results showed that the target genes of miR-214-3p in proliferation and estradiol synthesis are *Mfn2* and *NR5A1*, respectively.

**Conclusions:**

Our findings suggest that miR-214-3p plays an important role in the functional regulation of porcine GCs and therefore may be a target gene for regulating follicular development.

## Introduction

Granulosa cells (GCs), as the largest cell population in mature follicles, are the body’s primary source of estrogen and progesterone. The morphology and function of GCs are altered by primordial follicle growth initiation, proliferation, differentiation, atresia, ovulation, and luteum formation. GCs also can regulate the development of oocytes and follicles by secreting cytokines and hormones, which further affect female reproductive performance [1, 2]. Thus, the proliferation and hormone secretion of GCs are closely related to the growth and development of follicles [3].

Follicle development in the ovary requires recruitment, selection and dominance processes. The original follicles gradually develop into primary follicles, secondary follicles, antral follicles, and preovulatory follicles [4] accompanied by the transformation of GCs from a monolayer to a cubic shape of 2–3 layers, followed by multiple layers and cavities [5]. Follicle growth is, to this effect, inseparable from GCs proliferation [6].

There are three types of estrogen, the most active of which is estradiol [7]. During the synthesis of estradiol, FSH (follicle-stimulating hormone) receptors produced by GCs bind to FSH from the pituitary gland, which activates the FSH signaling pathway and increases the expression of related enzymes (e.g., CYP11A1, a cytochrome P450) and promotes estradiol synthesis [8, 9]. FSH can interact with receptors in the surface membranes of GCs, activate adenylyl cyclase and subsequently increase intracellular cAMP levels. The expression of aromatase (*CYP19A1*) corresponds to the increase of E_2_ secretion. In addition, StAR can transport the cholesterol from the outer to the inner mitochondrial membrane, where it is converted to pregnenolone by CYP11A1. Estradiol promotes the formation of follicles and gonadotropin receptors in the ovary [10, 11], inhibits the apoptosis of GCs [12], facilitates the formation of corpus luteum, and maintains the corpus luteum and regulates steroid synthesis.

MicroRNA (miRNA) is a short (20-24 nt) non-coding RNA, which mainly binds to the 3’UTR of the target genes’ mRNA sequence to stimulate degradation of mRNA, to regulate mRNA expression at the post-transcriptional level and inhibit its translation [13, 14]. Many previous studies have demonstrated that miRNA regulates the biological function of GCs by its targets. For example, in mouse GCs, miRNA-746-3p targets steroidogenic factor-1 (*SF-1*) to regulate 17β-estradiol synthesis [15]. MiR-202-5p induces apoptosis in goat GCs by targeting *TGFβR2* [14]. Another research proved that miR-1275 controls GCs apoptosis and estradiol synthesis by impairing *LRH-1/CYP19A1* axis [16]. However, certain phenotypes and mechanisms that other miRNAs regulate porcine ovarian GC proliferation and estradiol synthesis yet merit further research.

MiR-214 is transcribed from Dynamin 3 and forms a vertebrate-specific conserved cluster with miR-199 [17, 18]. Research on miR-214-3p tends to center on oncology, skeletal muscle development, adipogenesis, and similar applications [19–21]. Sequencing results from the ovarian tissue of Yorkshire pigs have shown that the expression of miR-214-3p in ovary tissues of high-yielding sows is higher than that in low-yielding sows [22]. Studies have also shown that miR-214 may regulate steroids by targeting low-density lipoprotein receptor genes in rat GCs [23]. We used Kyoto Encyclopedia of Genes and Genomes (KEGG) pathway and Gene Ontology (GO) analyses to find that miR-214-3p is involved in the physiological processes of cell proliferation and estradiol synthesis. In short, the literature suggests that miR-214 is involved in the biological functions of GCs. However, the specific effects of miR-214 on GCs remain unclear and are worth further analysis.

According to the above analysis, we suspect that miR-214-3p may be involved in the biological functions of GCs. In this study, we sought to detect whether miR-214-3p affects cell proliferation and estradiol synthesis by targeting functional genes in the GCs. The results presented here may provide new insight into the mechanisms by which miR-214-3p regulates biological functions of GCs.

## Materials and methods

### Identification of high-yielding and low-yielding sows

**Fig. 1:**
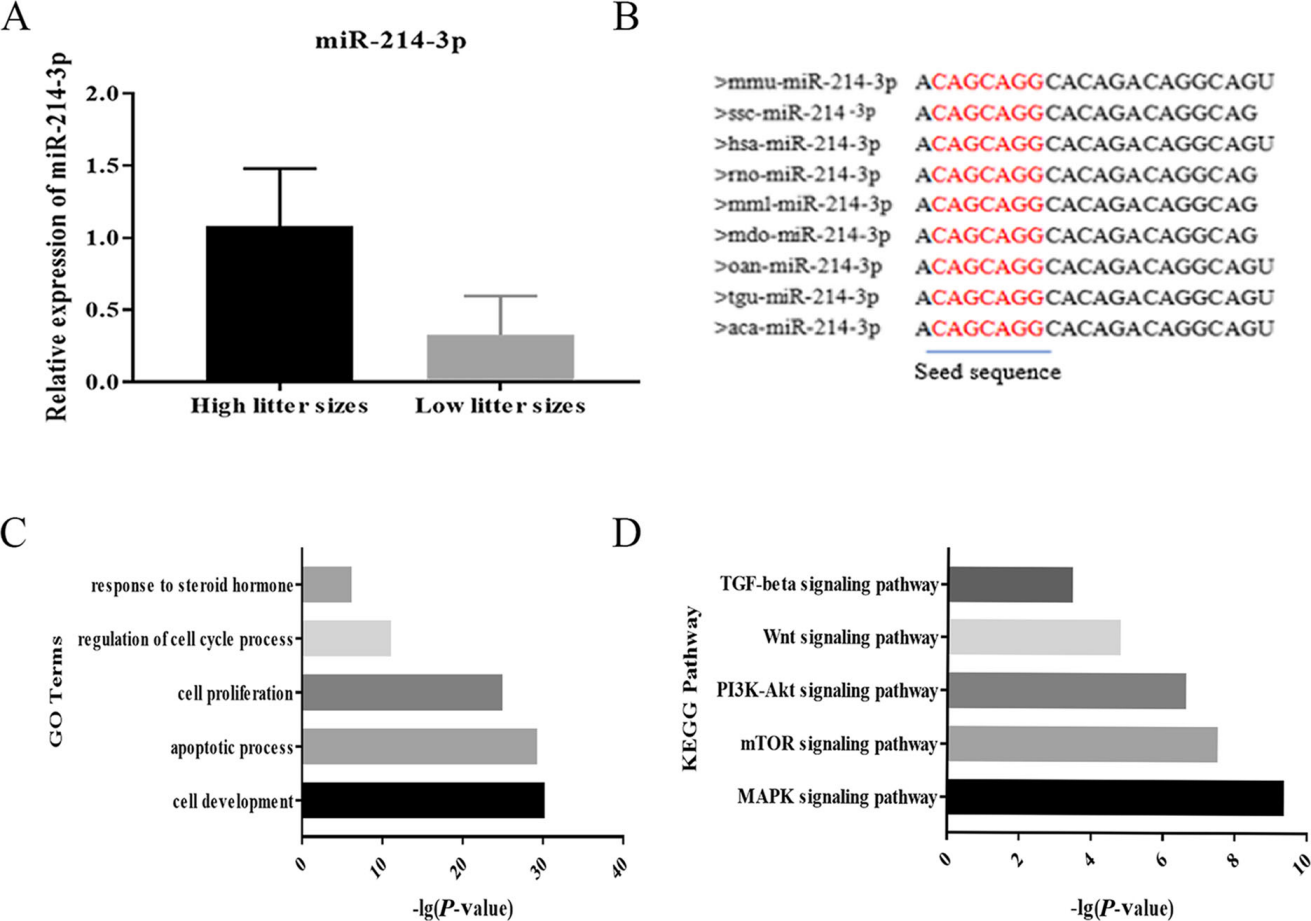
Expression level of miR-214-3p in porcine ovarian tissue (high-litter, low-litter) and bioinformatics analysis. **a** RT-qPCR analysis of miR-214-3p expression; **b** Sequence of mature miR-214-3p highly conserved across species; **c** GO term analysis of miR-214-3p; **d** KEGG pathway analysis of miR-214-3p

We collected and collated litter size records (a total of 8,657 parity) from Hanshiwei Food Ltd., Co. (Dahua, Guangxi, China) from 2016 to 2018 and used SPSS25.0 to perform normal distribution processing on the data. After normal transformation and testing, the total litter size (12.9 ± 2.17) was found to be approximately normally distributed, with a critical value of 15% right tail probability (14.7 head/litter) and a critical value of 15% left tail probability of (9.3 head/litter). Therefore, we defined the lower litter sizes as smaller litter sizes (SLS) below 9.3 head/litter and the higher yield groups above 14.7 head/litter as larger litter sizes (LLS). The ovarian tissues of three sows in each of the two groups were used as shown in Fig. 1a.

### Granulosa cells isolation and culture

Landrace ovaries (*n* = 20) from cyclic sows (*Sus scrofa*) were obtained immediately after slaughter, soaked in saline solution, and stored at 37 °C. The ovaries were shipped back to the laboratory within 2 h and were dissected and cleaned in thermostatic saline solution. The antral follicles (3–5 mm diameter) situated on the ovarian surface were punctured by needles to release the follicular fluid and flushed with Dulbecco’s modified Eagle’s medium/Nutrient Mixture F12 (DMEM/F12) medium (Cytiva, Buckinghamshire, England) containing 3% BSA, 1 IU FSH and 1 IU LH [24]. The culture medium with GCs and cumuluse oocyte complexes was filtered through a 70-mm cell strainer. The cumuluse oocyte complexes were filtered out and the filtrate with GCs was centrifuged at 1,000×*g* for 10 min. The GCs were then suspended with DMEM/F12 containing 3% BSA, inoculated in a cell culture well, and cultured in a cell incubator with 5% CO_2_ at 37 °C [3].

### Transfection of miRNA agomir and antagomir

An agomir is a type of specially labeled and chemically modified double-stranded microRNA which can regulate the biological function of a target gene by mimicking endogenous microRNA. An antagomir is a type of specially labeled and chemically modified single-stranded microRNA, designed based on the mature microRNA sequence, which can inhibit the expression of endogenous microRNA. The miR-214-3p agomir, antagomir, and respective nonspecific control (NC) materials used in this study were purchased from GenePharma (GenePharma, Shanghai, China) and were transfected into GCs with X-treme GENE HP DNA Transfection Reagent (Roche, Mannheim, Germany) at a final concentration of 50 nmol according to the manufacturer’s protocol. The medium was changed once after 24 h of transfection [21].

### RNA isolation and quantitative real-time PCR

Total RNA samples were isolated using Trizol (Takara, Otsu, Japan). The final concentrations were measured by NanoDrop 2000 (Thermo, Waltham, MA, USA). The cDNA was synthesized using a reverse transcription kit (Takara, Otsu, Japan). We used quantitative real-time PCR (RT-qPCR) for mRNA analysis. Every reaction was performed in triplicate with SYBR Premix (Vazyme, Nanjing, China) on a StepOne Real-Time PCR Machine (ABI, Carlsbad, CA, USA) [25]. The relative mRNA level was normalized to that of *Gapdh* and calculated using the 2^-∆∆Ct^ algorithm. The primer sequences we used for the RT-qPCR are listed in Table 1.

**Table 1:** Primer sequences used in this study (*Sus scrofa*)

Gene name	Forward	Reverse
Cyclin B	5’-AATCCCTTCTTGTGGTTA-3’	5’-CTTAGATGTGGCATACTTG-3’
Cyclin D	5’-TACACCGACAACTCCATCCG-3’	5’-GAGGGCGGGTTGGAAATGAA-3’
Cyclin E	5’-AGAAGGAAAGGGATGCGAAGG-3’	5’-CCAAGGCTGATTGCCACACT-3’
Star	5’-CGTTTAAGCTGTGTGCTGGG-3’	5’-TCCATGACCCTGAGGTTGGA-3’
Cyp11a1	5’-GGGCAACCCATTTCCTACCA-3’	5’-CGAGCACTGGTGGTACAGAC-3’
Cyp19a1	5’-TCCGCAATGACTTGGGCTAC-3’	5’-GCCTTTTCGTCCAGTGGGAT-3’
Mfn2	5’-AGCGGCTGCGGTTTATC-3’	5’-TCTATATGGCGATGCAGTTCA-3’
NR5A1	5’-CTGCCTCAAGTTCCTCATTCTC-3’	5’-GGTAGTGGCACAGGGTGTAATC-3’
Gapdh	5’-AGGTCGGAGTGAACGGATTTG-3’	5’-CCATGTAGTGGAGGTCAATGAAG-3’

### Western blot analysis

The cell total protein was isolated using RIPA (Applygen Technologies Inc., Beijing, China). Protease inhibitor (CWBIO, Shanghai, China) was added into the RIPA at a ratio of 1:100. After adding RIPA to the cell culture plate, we collected the cells and centrifuged (12,000 r/min) the material at 4 °C for 10 min [26]. Protein concentrations were measured on a Thermo Scientific Pierce BCA protein assay kit (Thermo Fisher, Massachusetts, USA) with 1/4 volume of 5 × loading buffer added to the supernatant. A total of 20 μL of protein was blotted using 10% SDS-polyacrylamide gel, then transferred to a polyethylene difluoride (PVDF) membrane (CST, Boston, MA, USA).

After blocking with 5% defatted milk for 2 h, the membranes were incubated overnight at 4 °C with antibodies (1:1,000) against StAR, CYP19A1, CYP11A1, Mfn2, NR5A1/SF-1 (Abcam, Cambridge, UK) and against cyclin B, cyclin D, cyclin E, CDK4 (Santa Cruz, TX, USA). The membrane HRP goat anti-mouse IgG, goat anti-rabbit IgG, and rabbit anti-goat IgG secondary antibodies (BOSTER, Wuhan, China) were diluted 1:3,000 according to the instructions and incubated for 1 h. Detection was performed using chemiluminescence Western blotting substrate (Santa Cruz, CA, USA) in Image Lab analysis software Image Lab™, (Bio-Rad, Berkeley, CA, USA).

### Flow cytometry

Porcine GCs were cultured in a 6-well culture plate at a density of 4 × 10^5^ per well. The cells were treated with miR-214-3p-agomir or antagomir for 48 h. The cells were digested with 0.25% trypsin and terminated with DMEM containing 10% FBS, then collected and fixed in cold 70% ethanol overnight at 4 °C [27]. The cells were then washed twice and stained with 50 mg/mL propidium iodide (PI) for 30 min. Finally, the cell cycles of the porcine subcutaneous preadipocytes were analyzed by flow cytometry (Becton Dickinson, Franklin Lakes, NJ, USA).

### EdU staining

GCs were seeded in 96-well plates at a concentration of 2 × 10^3^ per well. The GCs were then treated with miR-214-3p agomir and antagomir for 48 h and incubated with 50 μmol EDU (RiboBio, Guangzhou, China) for 2 h. The cells were washed twice with PBS, fixed with 4% paraformaldehyde for 30 min, neutralized with 2 mg/mL glycine for 5 min, then permeabilized with 0.5% Trixon-100 for 5 min. At the end of each step, the cells were washed twice with PBS for 5 min. According to the kit, the cells were incubated in a mixture of Reagents B, C, D, and E for 30 min. The cells were then washed three times with 0.5% Trixon-100, then twice with methanol. The nuclei were stained with Hoechst for 30 min. The stained cells were finally observed on a Nikon TE2000 microscope (Nikon, Tokyo, Japan) and the data were analyzed in Image J.

### Cell counting kit-8

Porcine GCs were seeded in 96-well plates with 2000 cells per well. After 48 h of rHhip treatment, 10 mL CCK8 reagent (Vazyme, Nanjing, China) was added into each well away from light, then the cells were incubated at 37 °C for 2–4 h. Finally, the plate absorbance was measured at 450 nm.

### Luciferase reporter assay

Luciferase reporter plasmids (psi-CHECK2) containing the wild-type 3’UTRs of *Mfn2/NR5A1* (WT-*Mfn2/NR5A1*) and mutant 3’UTRs of *Mfn2/NR5A1* (Mut-*Mfn2/NR5A1*) were obtained as manufactured by General Biosystems Co., Ltd. (General Biosystems, Anhui, China). HEK293T was seeded in a 48-well plate. X-treme GENE HP DNA Transfection Reagent was used to co-transfect the HEK293T cells with the wild-type or mutant 3’UTR luciferase reporter plasmids [28] and the miR-214-3p agomir or the negative control, respectively. The cells were harvested 24 h after transfection. Luciferase activities were measured on a Dual-Glo Luciferase AssaySystem (Promega; Madison, WI, USA) following the manufacturer’s instructions. Firefly luciferase was used as a normalization control.

### ELISA

E_2_ existing in the follicular fluid and medium supernatant was detected using a porcine E_2_ ELISA Kit (Nanjing Jiancheng Bioengineering Institute, Nanjing, China) operated according to the manufacturer’s instructions (tolerance within batch: CV < 10%; tolerance between batches: CV < 12%; sensitivity: 20–6,000 ng/L). The ELISA kit is coated with monoclonal antibodies and there is basically no cross reaction.

### Bioinformatics method

We performed a bioinformatics analysis using TargetScan, miRBase and miRTarBase. Many thousands of potential target genes were predicted. The common target gene associated with myogenes was predicted by at least these three programs. We also used KOBAS 3.0 to complete a Gene Ontology (GO) analysis and the Kyoto Encyclopedia of Genes and Genomes (KEGG) for further analysis.

### Statistical analysis

Statistical analyses were performed in GraphPad Prism 6 software. One-way analysis of variance (ANOVA) and a Newman-Keuls test were used to compare the groups. A paired Student’s test was used for comparison between any two groups. The data is presented here as the mean ± SEM of at least three independent experiments with statistical significance of * = *P* < 0.05; ** = *P* < 0.01.

## Results

### Biological characteristics of miR-214-3p

We detected the expression level of miR-214-3p in the ovarian tissue of Yorkshire × Landrace sows with high-litter and low-litter characteristics in this study. We observed a higher expression in high-litter sows than in low-litter sows (Fig. 1a). The mature sequence of miR-214-3p is highly conserved across multiple species (e.g., mouse, pig, human, rat) (Fig. 1b). We also performed GO analysis on the targets of miR-214-3p to find that it may indeed be involved in follicular growth processes such as cell proliferation and steroid synthesis (Fig. 1c). The TGF-beta and mTOR signaling pathways play important roles in the process of follicular growth. Our KEGG pathway analysis showed that miR-214-3p participates in these signaling pathways (Fig. 1d).

### miR-214-3p overexpression promotes granulosa cell proliferation

**Fig. 2:**
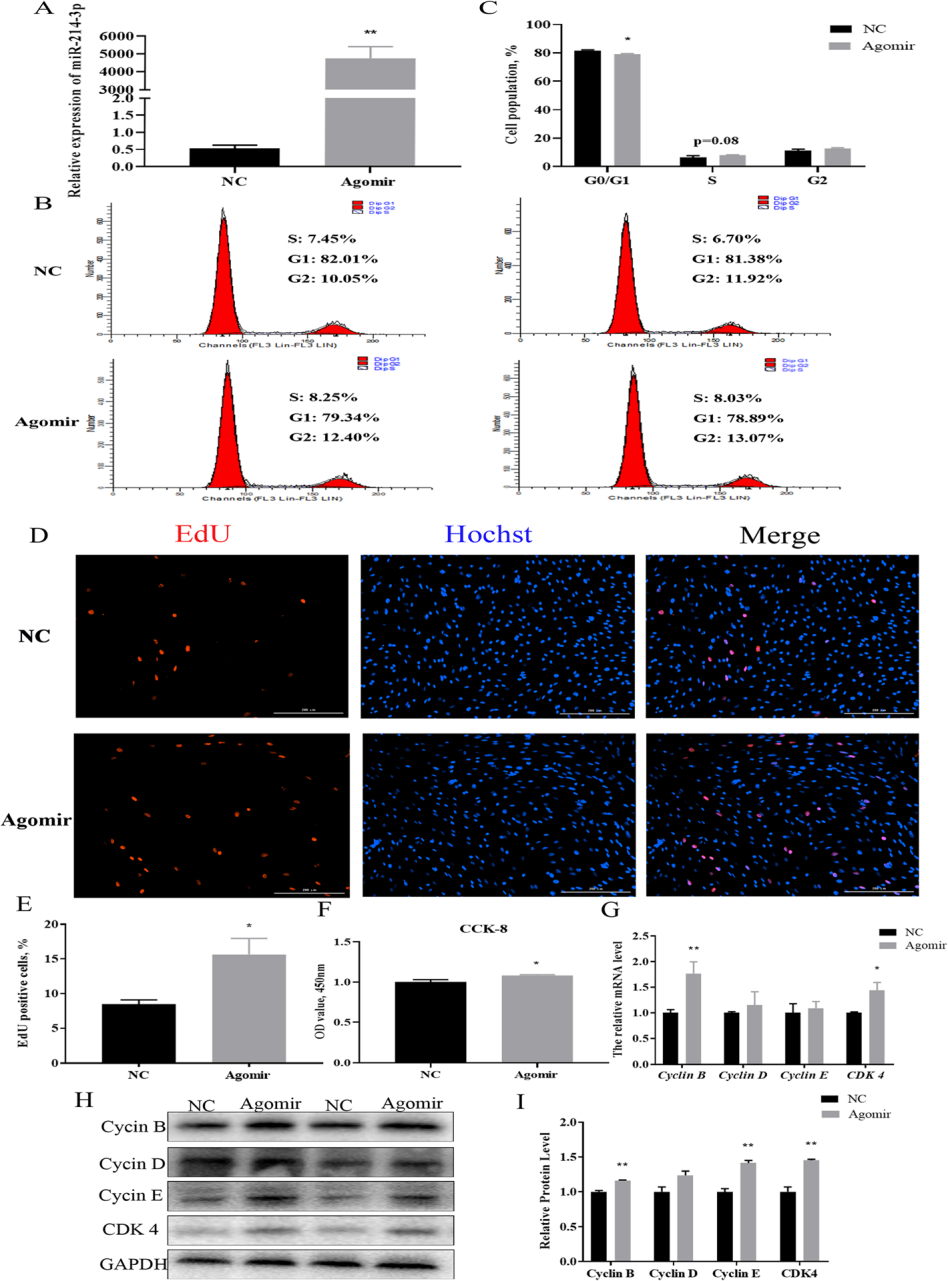
Overexpression of miR-214-3p promotes porcine GC proliferation. MiR-214-3p agomir or negative control (NC) transfected into cells harvested after 24 h. **a** Overexpression efficiency of miR-214-3p after transfection with miR-214-3p agomir compared to NC; **b** Flow cytometry determines cell percentages in different cycle phases; **c** Cell cycle analysis statistical results; **d** EdU staining assay of proliferous cell quantities. Positive cells stained by EdU (red) and total cell nucleus stained with Hoechst (blue); **e** Results presented as red/blue cell nuclei; **f** CCK-8 assay detects cell viability after 24-h transfection as absorbance value at 450 nm after incubation with 10% CCK-8 solution for 4 h; **g** RT-qPCR detects cell cycle genes, *Cyclin B, Cyclin E, Cyclin D, CDK4* after 24-h transfection; **h** Western blot analysis of cell cycle genes; **i** Quantification of Western blot analysis of Cyclin B, Cyclin D, Cyclin E, CDK4. Note: Data are mean ± SEM of three independent experiments; * *P* < 0.05, ** *P* < 0.01

In order to determine the effect of miR-214-3p on the proliferation of porcine ovarian GCs, we transfected the GCs samples with miR-214-3p agomir, antagomir, and the negative control. The expression of miR-214-3p increased significantly after transfection into agomir (Fig. 2a). Flow cytometry analysis indicated that miR-214-3p increased the percentage of S-phase cells (Fig. 2b,c). The EdU staining assay showed that the number of EdU labeled positive cells increased in the miR-214-3p agomir group, unlike in the negative control group (Fig. 2d,e). The CCK-8 assay also up-regulated cell viability (Fig. 2f). In addition, cell cycle-related genes (*Cyclin B, Cyclin E, and CDK4*) showed remarkably higher mRNA and protein levels but there was no such effect in cyclin D (Fig. 2g-i).

**Fig. 3:**
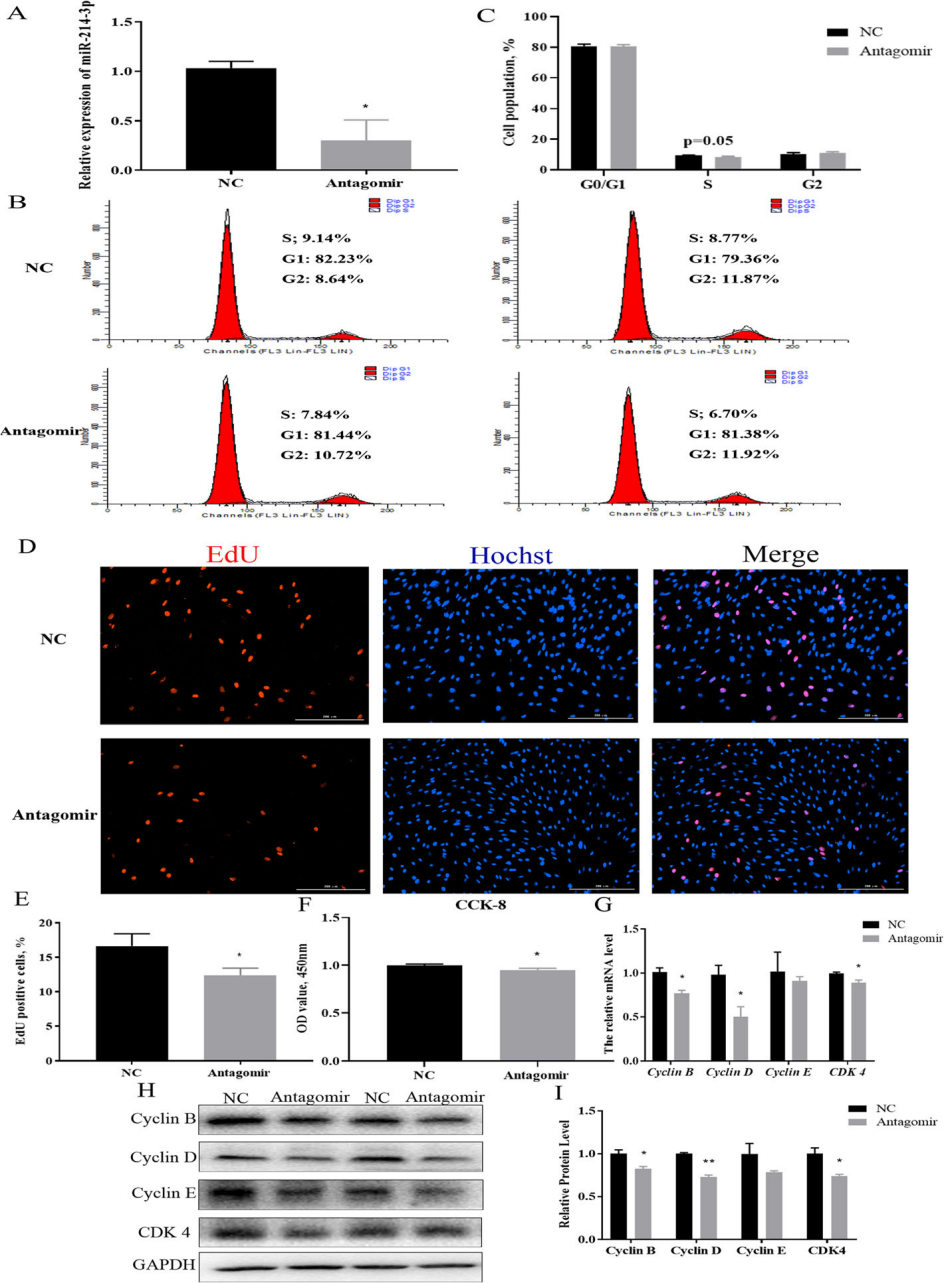
MiR-214-3p inhibitor inhibits porcine GC proliferation. MiR-214-3p antagomir or NC transfected into cells harvested after 24 h. **a** Knock-down efficiency of miR-214-3p after transfection with miR-214-3p antagomir compared to NC; **b** Flow cytometry determines cell percentages in different cycle phases; **c** Cell cycle analysis statistical results; **d** EdU staining assays proliferous cell quantities. Positive cells stained by EdU (red) and total cell nucleus stained with Hoechst (blue); **e** Results represented as red/blue cell nuclei percentages; **f** CCK-8 assay detects cell viability after 24-h transfection as absorbance value at 450 nm after incubation with 10% CCK-8 solution for 4 h; **g** RT-qPCR detects cell cycle genes, *Cyclin B, Cyclin E, Cyclin D, CDK4* after 24-h transfection; **h** Western blot analysis of cell cycle genes; (I) Quantification of Western blot analysis of Cyclin B, Cyclin D, Cyclin E, CDK4. Note: Data represent mean ± SEM of three independent experiments; * *P* < 0.05, ** *P* < 0.01

To further explore the effect of miR-214-3p on GC proliferation, we next treated the cells with antagomir-NC and antagomir. The expression of miR-214-3p in the treatment group was dramatically reduced below the negative control group (Fig. 3a). The flow cytometry results indicated down-regulation of the S-phase cells after suppressing the expression of miR-214-3p (Fig. 3b,c). Our EdU staining assay showed that inhibition of miR-214-3p can markedly decrease the number of EdU labeled positive cells (Fig. 3d,e). Our CCK-8 assay also verified the knock-down of miR-214-3p induced cell viability (Fig. 3f). RT-qPCR and Western blot data showed that miR-214-3p inhibition depressed the expression of cell cycle genes (Fig. 3g-i). In summary, miR-214-3p was found to promote GC proliferation.

### MiR-214-3p targets *Mfn2* in GCs

**Fig. 4:**
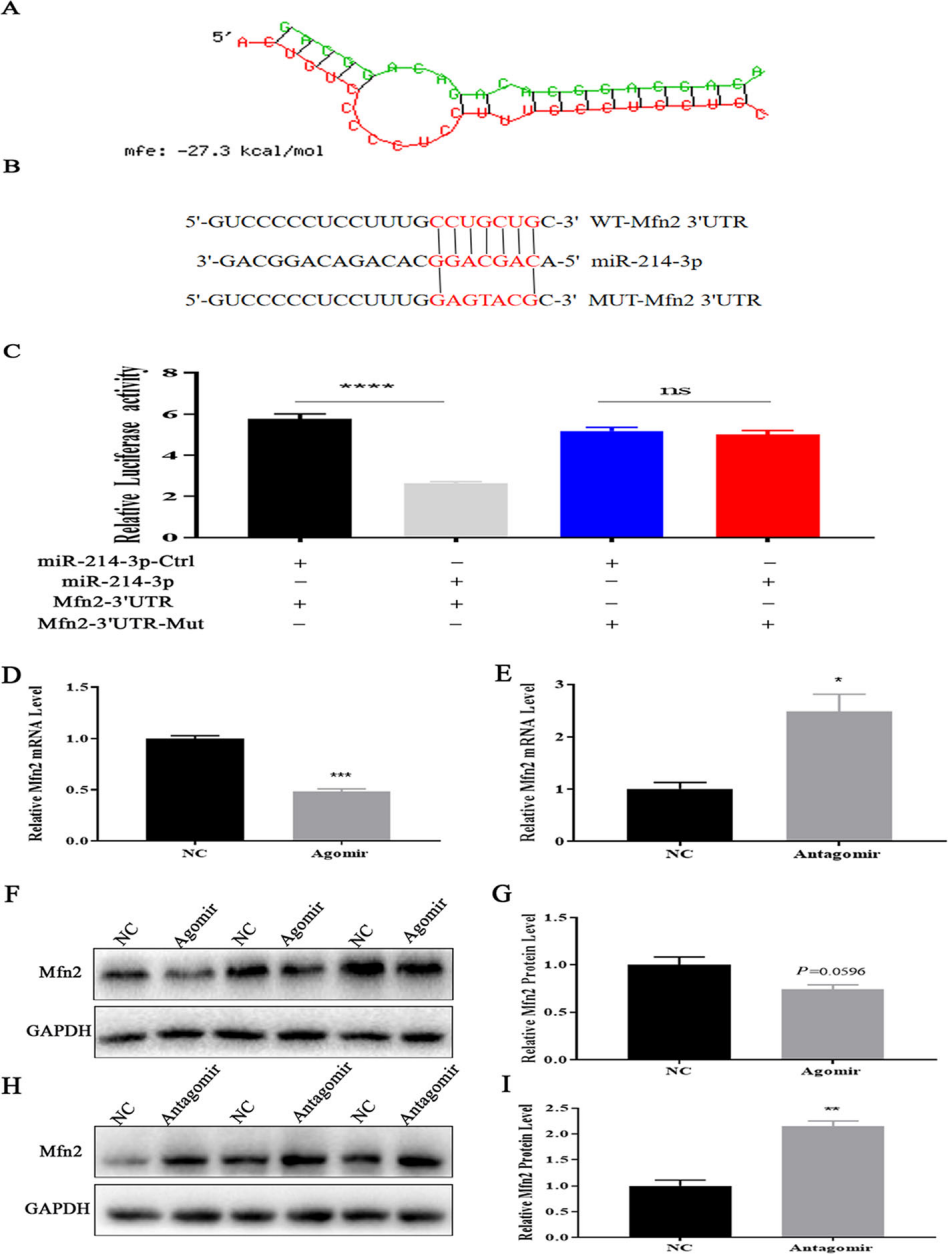
MiR-214-3p targets *Mfn2* during GC proliferation. **a** miR-214-3p binding site within the *Mfn2* 3′-UTR predicted by RNAhybrid; **b** Target site of miR-214-3p within porcine *Mfn2* mRNA 3’UTR and mutational site of *Mfn2* 3’UTR; **c** Dual luciferase assay by co-transfection of miR-214-3p agomir and wild-type vectors or mutant vectors. Relative luciferase activity represented by Renilla Luciferase/Firefly Luciferase (RLUC/FLUC); **d** Relative *Mfn2* mRNA expression levels after treatment with miR-214-3p agomir; **e** Relative *Mfn2* mRNA expression levels after treatment with miR-214-3p antagomir; **f** Western blot analysis of Mfn2 protein expression after treatment with miR-214-3p agomir; **h** Western blot analysis of Mfn2 protein expression after treatment with miR-214-3p antagomir; **g,i** Mfn2 protein level quantifications. Note: Data are mean ± SEM of three independent experiments. * *P* < 0.05, ** *P* < 0.01

The experiments described above showed that miR-214-3p can promote porcine GC proliferation (Figs. 2 and 3). To better understand the regulatory mechanism of this process, we used TargetScan7.2 and miRTarBase to predict potential target genes. We detected *Mfn2* as a candidate gene from thousands of target genes (Fig. 4a) and constructed wild-type *Mfn2* 3’UTR and mutant *Mfn2* 3’UTR dual luciferase reporter vectors accordingly (Fig. 4b). We found that the dual-luciferase activity of wild-type *Mfn2* 3’UTR and agomir co-transfected into GCs was higher than that of co-transfected wild-type *Mfn2* 3’UTR and NC, while mutant dual-luciferase activity with NC and agomir appears to have no effect (Fig. 4c).

Our RT-qPCR and Western blot data also suggest that Mfn2 mRNA and protein levels were reduced and increased, respectively, in the miR-214-3p agomir and antagomir groups (Fig. 4d-i). Altogether, our tests demonstrated that miR-214-3p promotes GC proliferation by directly targeting *Mfn2*.

### Correlation of miR-214-3p with GC estradiol synthesis

**Fig. 5:**
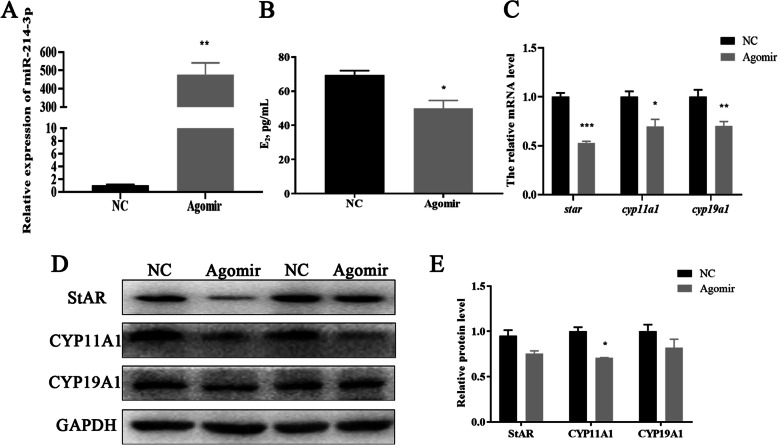
Overexpression of miR-214-3p inhibits porcine GC estradiol synthesis. MiR-214-3p agomir or NC transfected into cells harvested after 24 h. **a** Overexpression efficiency of miR-214-3p after transfection with miR-214-3p agomir compared to NC; **b** Estradiol concentration detected by ELISA. Culture supernatants collected 24 h after miR-214-3p agomir and NC treatment; **c** RT-qPCR detects E_2_ synthesis-related genes including *Star, Cyp11a1,* and *Cyp19a1* after 24-h transfection; **d** Western blot analysis of E_2_ synthesis-related genes; (E) Quantification of Western blot analysis of StAR, CYP11A1, CYP19A1. Note: Data are mean ± SEM of three independent experiments; * *P* < 0.05, ** *P* < 0.01

**Fig. 6:**
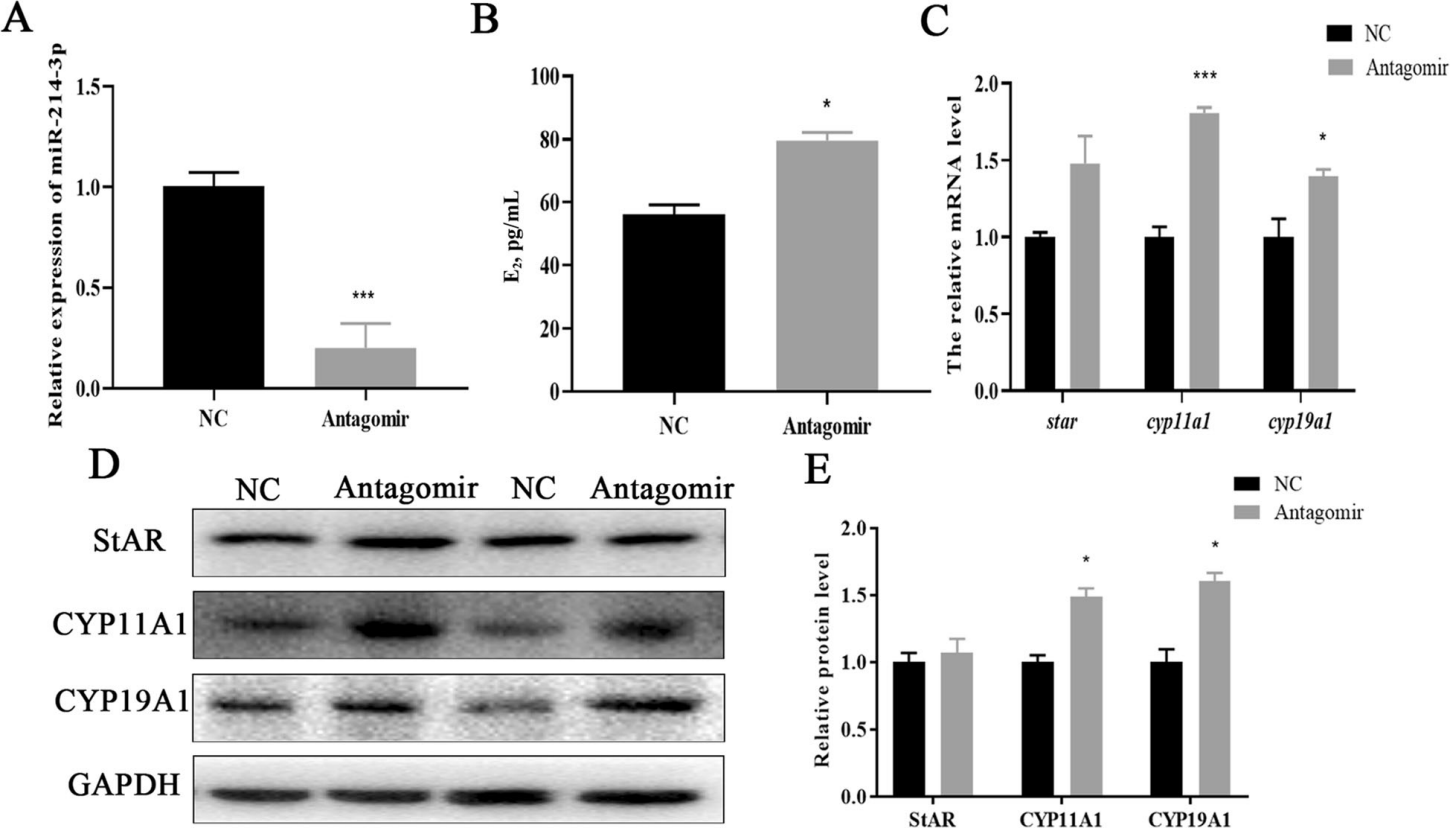
Inhibitor of miR-214-3p promotes porcine GC estradiol synthesis. MiR-214-3p agomir or NC were transfected into cells which were harvested after 24 h. **a** Overexpression efficiency of miR-214-3p after transfection with miR-214-3p antagomir compared to NC; **b** Estradiol concentration detected by ELISA. Culture supernatants collected 24 h after miR-214-3p antagomir and NC treatment; **c** RT-qPCR detects E_2_ synthesis-related genes including *Star, Cyp11a1,* and *Cyp19a1* after 24-h transfection; **d** Western blot analysis of E_2_ synthesis-related genes; **e** Quantification of Western blot analysis of StAR, CYP11A1, CYP19A1. Note: Data are mean ± SEM of three independent experiments; * *P* < 0.05, ** *P* < 0.01

One of the most important functions of GCs is the secretion of estradiol. We detected the E_2_ concentration in our culture medium accordingly. The expression of miR-214-3p increased or decreased sharply after transfection with agomir or antagomir (Figs. 5 and 6a). The ELISA results demonstrated that E_2_ concentration was markedly down-regulated or up-regulated in different treatment groups (Figs. 5 and 6b). E_2_ synthesis-related genes including *Star, Cyp11a1, and Cyp19a1* were also suppressed in mRNA and protein levels in the agomir group (Fig. 5c-e). The results in the antagomir treatment group were consistent with this (Fig. 6c-e). We infer that miR-214-3p inhibits GC estradiol synthesis.

### MiR-214-3p directly inhibits *NR5A1/SF-1* in GCs

*NR5A1* is also referred to as “steroidogenic factor 1” (*SF-1*) and is known to regulate estradiol synthesis by regulating the transcription of *Cyp11a1* and *Cyp19a1* genes via binding to the nuclear receptor motifs. To explore the mechanism by which miR-214-3p regulates estradiol synthesis, we forecasted the target genes of miR-214-3p with TargetScan7.2 and miRTarBase.

**Fig. 7:**
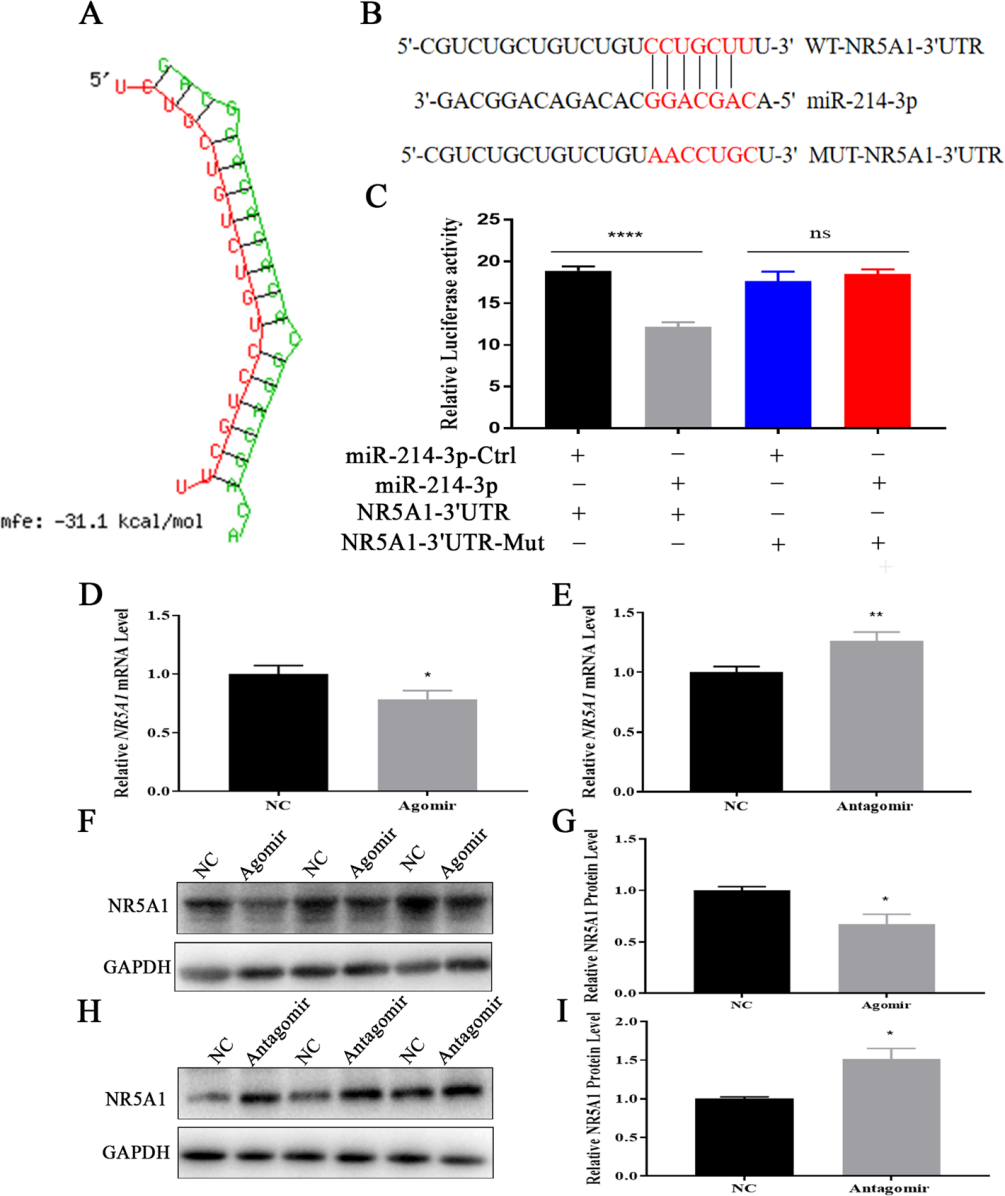
MiR-214-3p targets *NR5A1* during GC estradiol synthesis. **a** miR-214-3p binding site within *NR5A1* 3′-UTR predicted by RNAhybrid; **b** target site of miR-214-3p within porcine *NR5A1* mRNA 3’UTR and mutational site of *NR5A1* 3’UTR; **c** Dual luciferase assay via co-transfection of miR-214-3p agomir and wild-type vectors or mutant vectors. Relative luciferase activity represented by RLUC/FLUC; **d** Relative *NR5A1* mRNA expression levels after treatment with miR-214-3p agomir; **e** Relative *NR5A1* mRNA expression levels after treatment with miR-214-3p antagomir; **f** Western blot analysis of NR5A1 protein expression after treatment with miR-214-3p agomir; **h** Western blot analysis of NR5A1 protein expression after treatment with miR-214-3p antagomir; **g, i** Quantification of NR5A1 protein levels. Note: Data are mean ± SEM of three independent experiments; * *P* < 0.05, ** *P* < 0.01

Coincidentally, *NR5A1/SF-1* is one of the candidate target genes of miR-214-3p. This caught our attention over the course of our analysis, so we tested it specifically as a target gene of miR-214-3p (Fig. 7a). Similar to the results reported in Section 3.3, we constructed a dual luciferase reporter vector for assay (Fig. 7b), the assay revealed that miR-214-3p markedly suppressed the dual-luciferase activity (Fig. 7c). NR5A1/SF-1 mRNA and protein levels were also attenuated and increased in the miR-214-3p agomir and antagomir groups (Fig. 7d-i). These observations suggest that miR-214-3p inhibits GC estradiol synthesis by targeting *NR5A1/SF-1*.

## Discussion

In this study, we found that miR-214-3p plays an important role in GC proliferation and estradiol synthesis. Specifically, miR-214-3p promotes GC proliferation and inhibits estradiol synthesis. Our findings represent workable information regarding the regulation of GCs functions by miR-214-3p. GC functions such as proliferation and estradiol synthesis are affected by many regulatory factors [29–31]; miRNA plays an important part in these processes [32, 33] and miR-214-3p is expressed to greatest extent in porcine ovarian tissue among other body tissues [23]. We used high-yield (> 14.7 head/litter) and low-yield (< 9.3 head/litter) ovary tissues of Yorkshire×Landrace to verify that miR-214-3p is expressed higher in high-yield sows but not only in Yorkshire [22], which indicates that miR-214-3p is important for reproduction. Bioinformatics analysis and conservative prediction also indicate that miR-214-3p plays a role in regulating GC function.

Our experimental results further indicate that miR-214-3p promotes proliferation by upregulating the mRNA and protein levels of Cyclin B, Cyclin D, Cyclin E, and CDK4 (Figs. 2 and 3g-i). *Cyclin B* is a marker of immunohistochemical proliferation [34] and CDK4 is a kinase that regulates the transition from the G1 to S phases of the cell cycle [35]. We found that due to miR-214-3p agomir and antagomir, compared to our NC, Cyclin B and CDK4 had the most significant differential expression of mRNA and protein levels. Flow cytometry, EdU staining, and CCK-8 assays also proved that miR-214-3p promotes proliferation in GCs (Fig. 2 and Fig. 3), which is consistent with previous research results. For example, miR-214-3p regulates the proliferation of breast cancer cells by targeting survivin protein [36] and can promote smooth muscle cell proliferation [37],

*Mfn2* is regarded as a proliferation inhibitor because it can limit the expression of Cyclin D protein to inhibit the proliferation process [38]. By RNAhybrid prediction, miR-214-3p binds to the 3′-UTR region of *Mfn2* (Fig. 4a). Accordingly, *Mfn2* can be used as a candidate target gene of miR-214-3p. In the present study, we found that miR-214-3p can repress the mRNA and protein levels of Mfn2 (Fig. 4d-i). This indicates that *Mfn2* is a direct target gene of miR-214-3p via dual-luciferase reporter assay (Fig. 4c) and that *Mfn2* can perform as a target gene for miR-214-3p to regulate cell proliferation. These results are consistent with previous reports, for instance, where Feng et al. [39] reported that miR-93 regulates vascular smooth muscle cell proliferation by targeting *Mfn2*. Additionally, miR-497 promotes cardiomyocyte proliferation by downregulating the expression of *Mfn2* [40].

There have been no such results regarding the synthesis of estradiol by miR-214-3p published previously. Our findings suggest, however, that miR-214-3p does inhibit the synthesis of estradiol (Fig. 5 and Fig. 6). During the synthesis of E_2_, StAR can transport cholesterol from the outer to the inner mitochondrial membrane, where it is converted to pregnenolone by CYP11A1 [41]. Aromatase (*CYP19A1*) in GCs transforms testosterone into estradiol [41, 42]. We found that miR-214-3p attenuated the transcription and translation levels of Star, Cyp11a1, and Cyp19a1 (Figs. 5 and 6c-e). These results enrich the existing knowledge of miR-214-3p in terms of the regulation of GC functions.

In order to further study the molecular mechanism of miR-214-3p regulating E_2_ synthesis in GCs, we selected *NR5A1/SF-1* as the target gene because it can bind to SF-1 response elements on the promoter of target genes such as *Star, Cyp11a1,* and *Cyp19a1* to regulate their transcription activity [43, 44]. *NR5A1/SF-1* also is present in fetal and adult steroidogenic tissues and participates in the regulation of ovarian function [45]. Therefore, *NR5A1/SF-1* may play an important role in E_2_ synthesis. Our results proved that miR-214-3p attenuates the mRNA and protein levels of NR5A1/SF-1 (Fig. 7d-i), which suggests that *NR5A1/SF-1* may be a target gene of miR-214-3p in GCs.

Our double luciferase reporter assay indicates that *NR5A1/SF-1* is the direct target gene of miR-214-3p (Fig. 7c). These data suggest that miR-214-3p inhibits E_2_ synthesis through *NR5A1/SF-1* in GCs. It is worth noting that many previous researchers have reached conclusions consistent with ours. For example, in mouse ovaries, miR-320 and miR-764-3p regulate estradiol synthesis by targeting *SF-1* [15, 46, 47].

## Conclusions

**Fig. 8:**
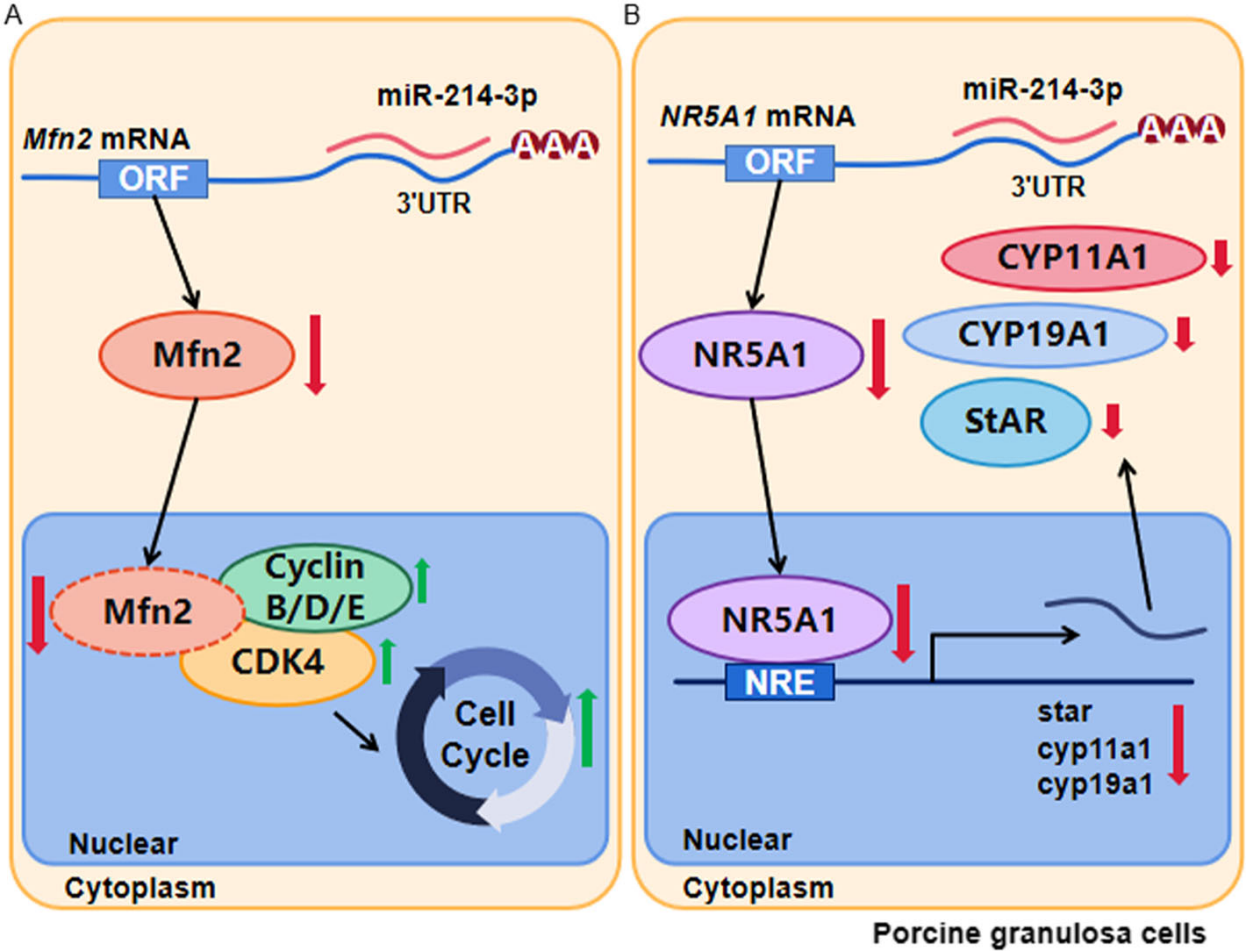
Schematic diagram of miR-214-3p regulation on porcine GC proliferation and estradiol synthesis. **a** MiR-214-3p promotes porcine GC proliferation by targeting Mfn2. **b** MiR-214-3p inhibits GC estradiol synthesis by targeting NR5A1. Note: Green upward arrows indicate promotion of a given process; red downward arrows indicate that this process is inhibited

In summary, as shown in Fig. 8, our results show that miR-214-3p promotes GC proliferation by targeting *Mfn2* and inhibits GC estradiol synthesis by targeting *NR5A1/SF-1*. The results presented here may provide workable insight into regulating the GCs functions, follicular growth and development.

## Data Availability

The data sets used and analysed during the current study are available from the corresponding author on reasonable request.

## Abbreviations

GCs: Granulosa cells
StAR: Steroidogenic acute regulatory protein
CYP11A1: Cytochrome P450 family 11 subfamily A member 1
CYP19A1: Aromatase
Cyclin B: Cell cycle protein B
Cyclin D: Cell cycle protein D
Cyclin E: Cell cycle protein E
CDK4: Cyclin-dependent kinase 4
mmu: *Mus musculus*
ssc: *Sus scrofa*
hsa: *Homo sapiens*
rno: *Rattus norvegicus*
mml: *Macaca mulatta*
mdo: *Monodelphis domestica*
oan: *Ornithorhynchus anatinus*
tgu: *Taeniopygia guttata*
aca: *Anolis carolinensis*
